# New Strategies in Tuberculosis Prediction: TB-CAST, an Explainable Baseline Score for Two-Month Sputum Culture Positivity

**DOI:** 10.3390/life16081384

**Published:** 2026-08-21

**Authors:** Marinela-Alina Miulescu, Victor-Ionel Grecu, Liliana Streba, Cristina Călărașu, Patricia-Mihaela Rădulescu, Costin-Teodor Streba

**Affiliations:** 1 Doctoral School, University of Medicine and Pharmacy of Craiova, 200349 Craiova, Romania; alyna_rai@yahoo.com; 2 Leamna Pneumology Hospital, 207129 Leamna, Romania; cristina.calarasu@umfcv.ro (C.C.); costin.streba@umfcv.ro (C.-T.S.); 3 “Victor Babeș” Infectious Diseases and Pneumophthisiology Hospital, 200515 Craiova, Romania; grecuvictorionel@yahoo.com; 4 Department of Oncology and Palliative Care, University of Medicine and Pharmacy of Craiova, 200349 Craiova, Romania; 5 Department of Pulmonology, University of Medicine and Pharmacy of Craiova, 200349 Craiova, Romania; patricia.radulescu@umfcv.ro

**Keywords:** tuberculosis, two-month culture status, clinical prediction score, machine learning, cross-validation, SHAP, mobile health

## Abstract

**Background:** Early identification of patients who will remain sputum-culture-positive at two months could support risk-adapted tuberculosis (TB) monitoring. We developed a compact baseline score from routine admission data measured before anti-TB therapy. **Methods:** The cohort comprised 155 adults (62 culture-positive and 93 culture-negative at two months). Random Forest ranking and correlation clustering were used as exploratory selection steps. The complete development pipeline was evaluated by repeated fully nested cross-validation; a secondary conditional analysis held the full-cohort-selected candidate set fixed. **Results:** The fully nested pipeline yielded a mean test-fold AUC of 0.70 ± 0.09 (fold-level percentile interval 0.53–0.85). Conditional on full-cohort predictor preselection, mean test-fold AUC was 0.74 ± 0.08, and the patient-level mean repeated out-of-fold score AUC was 0.75 (bootstrap 95% CI 0.67–0.83). At provisional thresholds of ≥2, ≥3 and ≥4 points, conditional procedure sensitivity/specificity were 0.90/0.39, 0.80/0.60 and 0.57/0.74, respectively. The conditional repeated out-of-fold Brier score was 0.200, with calibration intercept approximately 0.00 and slope 0.74. **Conclusions:** TB-CAST is an exploratory score with moderate internal discrimination. The fully nested result is the primary internal estimate for the complete data-driven pipeline; the conditional results and provisional thresholds require prospective external validation before clinical use.

## 1. Introduction

### 1.1. Global Context

Pulmonary tuberculosis (TB) remains a major global public health concern, especially in low- and middle-income countries, despite large-scale interventions such as DOTS and the End TB Strategy [1]. Drug-resistant TB and TB/HIV coinfection continue to complicate global control [2,3], while TB also imposes substantial long-term health and household financial burdens [4,5]. Within the European Union/EEA, Romania has consistently reported one of the highest tuberculosis notification rates, several-fold above the EU/EEA average [6]. According to the WHO Global Tuberculosis Report 2023, an estimated 10.6 million people developed TB in 2022, including 410,000 rifampicin-resistant or multidrug-resistant cases [3].

### 1.2. Treatment Monitoring and Its Limitations

The standard six-month regimen is generally effective but is undermined by adverse effects and long duration. The WHO 2021 guidelines define MDR-TB as resistance to both isoniazid and rifampicin, and pre-XDR-TB as MDR-TB with additional fluoroquinolone resistance [7]. Sputum culture conversion at two months is a widely used early marker of treatment response, yet its individual-level prognostic accuracy is modest; in MDR-TB, later conversion (4–6 months) correlates more strongly with final outcomes [8]. This has motivated complementary strategies, including host response biomarker panels [9], molecular assays such as Xpert MTB/RIF Ultra and Xpert MTB/XDR [10,11], and next-generation sequencing [12]. Combining such tools with routinely available clinical predictors may allow earlier individualised risk stratification.

### 1.3. Machine Learning and Study Aim

Machine learning methods, particularly tree-based models, have shown value across clinical prediction tasks [13,14,15,16,17], and multiparametric scores integrating clinical, haematological and radiological data have been proposed for TB [18,19,20,21]. Related tree-based and other machine-learning approaches have been applied to drug-resistance classification and clinical outcome prediction in infectious diseases, including TB [22,23,24,25]. A recurring weakness is that cut-points and weights are often selected and evaluated in the same data, producing optimistic performance estimates [26]. This prospective study was conducted from July 2022 to June 2024 at Leamna Pneumology Hospital in the Oltenia region of Romania. Using baseline data obtained before anti-TB therapy, we aimed to derive a compact score for two-month culture positivity and to quantify its internal validation performance, calibration and clinical utility profile. Exploratory Random Forest screening and correlation clustering were used to define a candidate set; subsequent cut-point and weight estimation were repeated within training folds. The resulting score is intended as a transparent candidate for future external validation and possible implementation as a paper or digital tool.

## 2. Materials and Methods

### 2.1. Study Population and the Treatment-Naïve Baseline

**Figure 1:**
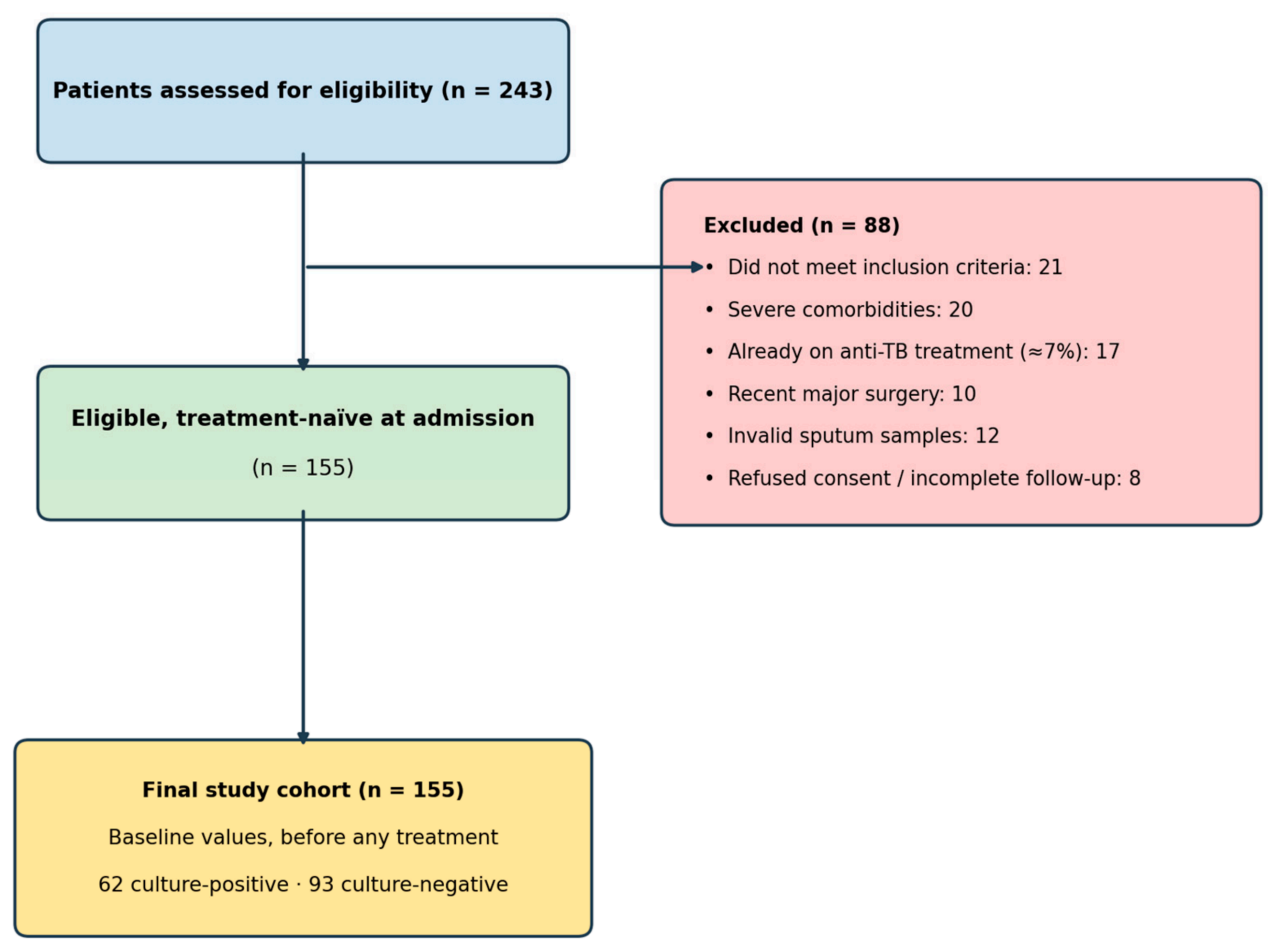
Study selection flowchart. Of 243 patients assessed, 88 were excluded—including approximately 7% (*n* = 17) who had already started anti-TB treatment—leaving 155 treatment-naïve patients whose baseline admission data formed the analysis cohort.

We conducted a single-centre prospective observational study of 155 adult inpatients with microbiologically confirmed pulmonary TB. Eligible patients were at least 18 years old, HIV-negative, and able to give written informed consent (Figure 1). A defining feature of this cohort is that every recorded value is a baseline measurement obtained on admission, before the initiation of any anti-TB or rebalancing therapy. This criterion is central to the study: because the endpoint to be predicted is two-month culture status, using pre-treatment values avoids incorporation (target leakage) bias and yields a genuine treatment initiation score. Accordingly, patients who had already started anti-TB treatment at the time of assessment—approximately 7% of those screened (*n* = 17)—were excluded.

**Endpoint definition.** Two-month culture status was determined on a sputum specimen collected 8 weeks (±2 weeks) after treatment initiation. Mycobacterial culture used the automated liquid BD BACTEC MGIT 960 system (Becton, Dickinson and Company, Sparks, MD, USA), with Löwenstein–Jensen solid medium; growth of Mycobacterium tuberculosis defined a positive culture, and persistent positivity denoted a positive culture at this visit irrespective of colony count. Laboratory staff performing and reading cultures were unaware of the baseline predictor values. Contaminated cultures were repeated when material allowed; patients without an evaluable two-month culture were not part of the analysed cohort. Baseline disease was microbiologically confirmed in every patient. The archived Xpert field was coded as MTB detected, not detected or not performed; it did not contain rifampicin resistance information. Phenotypic drug susceptibility results, treatment regimens administered during follow-up and adherence were not available in the locked analytic dataset; resistance- and regimen-stratified analyses were therefore not feasible (Section 4.6).

HIV-positive patients were excluded a priori to obtain a more homogeneous cohort, because HIV-related immunosuppression can alter both the clinical presentation of TB and the dynamics of culture conversion. The locked analytic dataset did not retain a validated rifampicin resistance or phenotypic drug susceptibility classification, and the available legacy source fields did not permit reliable reconstruction of MDR/RR or XDR status. We therefore do not classify the analysed cohort by resistance category and did not perform resistance- or regimen-stratified analyses. The endpoint was sputum culture status at two months: 62 patients (40.0%) were culture-positive and 93 (60.0%) were culture-negative at the two-month assessment.

All consecutive eligible patients during the predefined recruitment period were included; no a priori sample size calculation was performed. With 62 outcome events and eight retained score components, the study was treated as exploratory, and external validation was considered essential before clinical use.

### 2.2. Data Collection

On admission, each patient underwent a standardised clinical, laboratory and radiological evaluation, all performed before treatment. Recorded data comprised demographics and lifestyle factors; a complete blood count with differential (leukocytes, neutrophils, lymphocytes, eosinophils, monocytes, platelets, mean corpuscular volume [MCV], and red-cell distribution width–standard deviation [RDW-SD]); liver and kidney function tests; fasting glucose; and uric acid. Baseline thoracic imaging reports were abstracted into three independent structured variables: cavity number, maximum cavity size, and predominant radiological pattern. For reporting purposes, the archived pattern labels are operationally described as follows: infiltrative–nodular, infiltrative change with nodular opacities as the dominant morphology; infiltrative–ulcerative, an infiltrative/destructive (“ulcerative”) appearance recorded as the dominant pattern but not defined by the separate cavity count field; cavitary, cavitation as the dominant morphology; and mixed, coexistence of multiple morphologies without a single non-mixed dominant category. These source report categories were not algorithmically derived from, or logically constrained by, the separate cavity number or cavity size variables. The locked analytic dataset and legacy SPSS file retain one final radiological coding per patient but no imaging modality indicator or separate CT/radiograph ratings. Consequently, modality-specific counts and a formal CT–radiograph harmonisation analysis cannot be reconstructed reliably from the archived data. Images were reviewed independently by two experienced radiologists blinded to culture results, with disagreements resolved by consensus. Records were double-entered and cross-checked against source reports. Because complete baseline laboratory and imaging data were an inclusion requirement, no missing values were present for the 52 analysed variables and no imputation was performed. Before the final rerun, categorical value labels in the Python dataset were reconciled against the original SPSS value label dictionary. All 155 analytic records matched uniquely on laboratory values and outcome; the numeric codes used in the models were unchanged, and the correction concerned the English radiological labels.

### 2.3. Statistical Analysis

Analyses were performed in Python 3.12.3 using pandas 3.0.2, NumPy 2.4.4, SciPy 1.17.1, scikit-learn 1.8.0, stats models 0.14.6, SHAP 0.52.0 and matplotlib 3.10.8; descriptive results were cross-checked in SPSS 26.0. The local explanation used a deterministic weighted linear surrogate following the LIME framework. Continuous variables are reported as mean ± standard deviation when analysed with Welch’s *t*-test and as median [interquartile range] when analysed with the Mann–Whitney U test; categorical variables are reported as counts and percentages. Normality was assessed with the Shapiro–Wilk test; between-group comparisons used Welch’s *t*-test when the normality criterion was met and the Mann–Whitney U test otherwise. Categorical variables were compared with the chi-square test, or Fisher’s exact test for 2 × 2 tables. For Fisher tests, odds ratios compare category 1/present with category 2/absent for two-month culture positivity. Associations are expressed as crude and mutually adjusted odds ratios (OR) with 95% confidence intervals (CI). Analyses were exploratory; two-sided *p* < 0.05 was used without multiplicity adjustment, and *p*-values were not used as the sole basis for predictor retention. Calibration was summarised with the Brier score, calibration intercept and calibration slope.

### 2.4. Development of the Data-Driven Score

Score construction comprised a derivation stage followed by internal validation. Candidate selection began with a defined pool of 16 continuous biomarkers and the radiological variables. Continuous candidates were dichotomised at training-data cut-points maximising Youden’s J, and the resulting indicators plus categorical cavity and radiological pattern terms were entered into L2-penalised logistic regression (scikit-learn inverse regularisation parameter C = 0.5). A fixed scale of 0.5 log-odds units per point was applied consistently across the revised rerun; coefficients were divided by 0.5 and rounded to the nearest integer. Components rounding to zero were omitted from the displayed score. The patient’s total is the sum of the points for the features present.

Candidate selection rules. To make the exploratory selection reproducible, the revised analysis used the following rule, fixed before the complete rerun. A 300-tree Random Forest was fitted to the defined continuous-biomarker pool plus the radiological indicators. The eight continuous biomarkers with the highest Gini importance were retained and grouped by average-linkage hierarchical clustering on the Spearman correlation distance (1 − |ρ|), with the tree cut at a distance of 0.6 (grouping variables with |ρ| > 0.4). Within each cluster, the variable with the highest univariate AUC was retained; among the cluster representatives, the three with the highest univariate AUC were selected to preserve a three-biomarker score. Applied to the full derivation cohort, this rule selected leukocytes, RDW-SD and monocytes. In the conditional validation, this full-cohort candidate set was held fixed; in the fully nested sensitivity analysis, the complete selection rule was repeated inside each training fold. A separate all-variable Random Forest fitted on a single stratified 70:30 split provided the descriptive held-out AUC (0.63) and permutation importance estimates.

### 2.5. Validation and Explainability

Performance was evaluated with 10 repeated stratified 5-fold internal validation splits. Within every training fold, the three continuous cut-points, penalised logistic coefficients, integer points and a logistic mapping from total score to predicted probability were re-estimated and then applied to the untouched test fold. Because the initial exploratory screening and cluster-based candidate selection were performed once in the full derivation dataset, this analysis estimates performance conditional on the selected predictor set and is not described as fully nested validation. Discrimination was summarised as the mean and standard deviation of test-fold AUCs; additionally, each patient’s 10 out-of-fold scores were averaged, and a patient-level bootstrap (2000 resamples) was used for the AUC confidence interval and for percentile intervals for the Brier score, calibration intercept and calibration slope. Fixed thresholds of 2, 3 and 4 points were assessed using mean sensitivity, specificity, positive predictive value and negative predictive value across the 10 repeated partitions. Calibration used the averaged repeated out-of-fold probabilities, and decision curve analysis was treated as exploratory. Apparent and bootstrap optimism-corrected AUCs of the final derivation model were reported as secondary analyses. SHAP and a LIME-style local weighted surrogate were applied post hoc to the final derivation model for descriptive explainability and were not used as evidence of validation.

### 2.6. Ethics

The study followed the Declaration of Helsinki and was approved by the Institutional Review Board of Leamna Pneumology Hospital (approval 113/19.07.2021). Written informed consent was obtained from all participants, and patient confidentiality was safeguarded throughout.

## 3. Results

### 3.1. Baseline Characteristics at Admission

All results describe values recorded at admission, before any treatment was started. The 155 patients had a mean age of 51.3 years and were predominantly male (78.7%). Age (52.1 vs. 50.8 years, *p* = 0.537), sex (79.0% vs. 78.5% male, *p* = 1.000) and body mass index (median 19.1 vs. 19.4 kg/m^2^, *p* = 0.165) were similar between the two outcome groups in this cohort.

### 3.2. Univariate Analysis

Among the admission laboratory parameters, three markers of systemic inflammation differed significantly between groups (Table 1). Culture-positive patients had higher median leukocyte counts (10,390 [8638–11,865] vs. 8400 [6490–11,030]/µL; p = 0.001), neutrophil counts (7450 [5713–9360] vs. 6160 [4380–7950]/µL; p = 0.003) and platelet counts (326,500 [272,500–420,500] vs. 283,000 [227,000–352,000]/µL; p = 0.036). RDW-SD, monocytes and eosinophils were numerically higher in the positive group but were not statistically significant as continuous variables. Their subsequent inclusion in the candidate score reflected the predefined multivariable derivation procedure rather than univariate *p*-values alone.

The clinical and imaging features assessed on baseline thoracic imaging showed the strongest univariate associations (Table 2). Predominant radiological pattern (*p* = 0.0005), number of cavities (*p* = 0.0007), lymphadenopathy (*p* = 0.0015), cavity size (*p* = 0.0033) and extent of lung involvement (*p* = 0.0087) differed between groups. A history of tumours, dry cough and productive cough approached but did not cross the prespecified significance threshold.

Radiological coding. Cavity number, cavity size and predominant radiological pattern were coded as distinct variables. The original SPSS dictionary defined pattern codes as infiltrative–nodular (code 1; *n* = 17), infiltrative–ulcerative (code 2; *n* = 26), cavitary (code 3; *n* = 11), and mixed (code 4; *n* = 101; reference). The full three-way cross-tabulation of pattern, cavity number and cavity size is provided in Supplementary Table S1. Two source-coded cavity number/cavity size discordances were identified: one infiltrative–ulcerative record was coded as no cavities by cavity number but <10 mm by cavity size, and one mixed-pattern record was coded as no cavities by cavity number but >25 mm by cavity size. The source imaging detail needed to adjudicate these two records is not retained in the locked analytic dataset, so the primary analysis preserves the original source-coded values. In a sensitivity harmonisation that set cavity size to “no cavities” for these two records, all rounded integer point assignments were unchanged. Conditional mean fold AUC changed from 0.739 to 0.738, patient-level repeated out-of-fold score AUC from 0.754 to 0.750, and Brier score from 0.200 to 0.201; threshold-specific sensitivity or specificity changed by no more than 0.01 (Supplementary Table S4). The discrepancy therefore did not materially alter the score structure or conditional performance estimates. The previous correction to the English radiological pattern labels did not alter the underlying numeric codes. Formal inter-reader agreement before consensus was not quantified.

### 3.3. Exploratory Feature Importance and Redundancy Reduction

**Figure 2:**
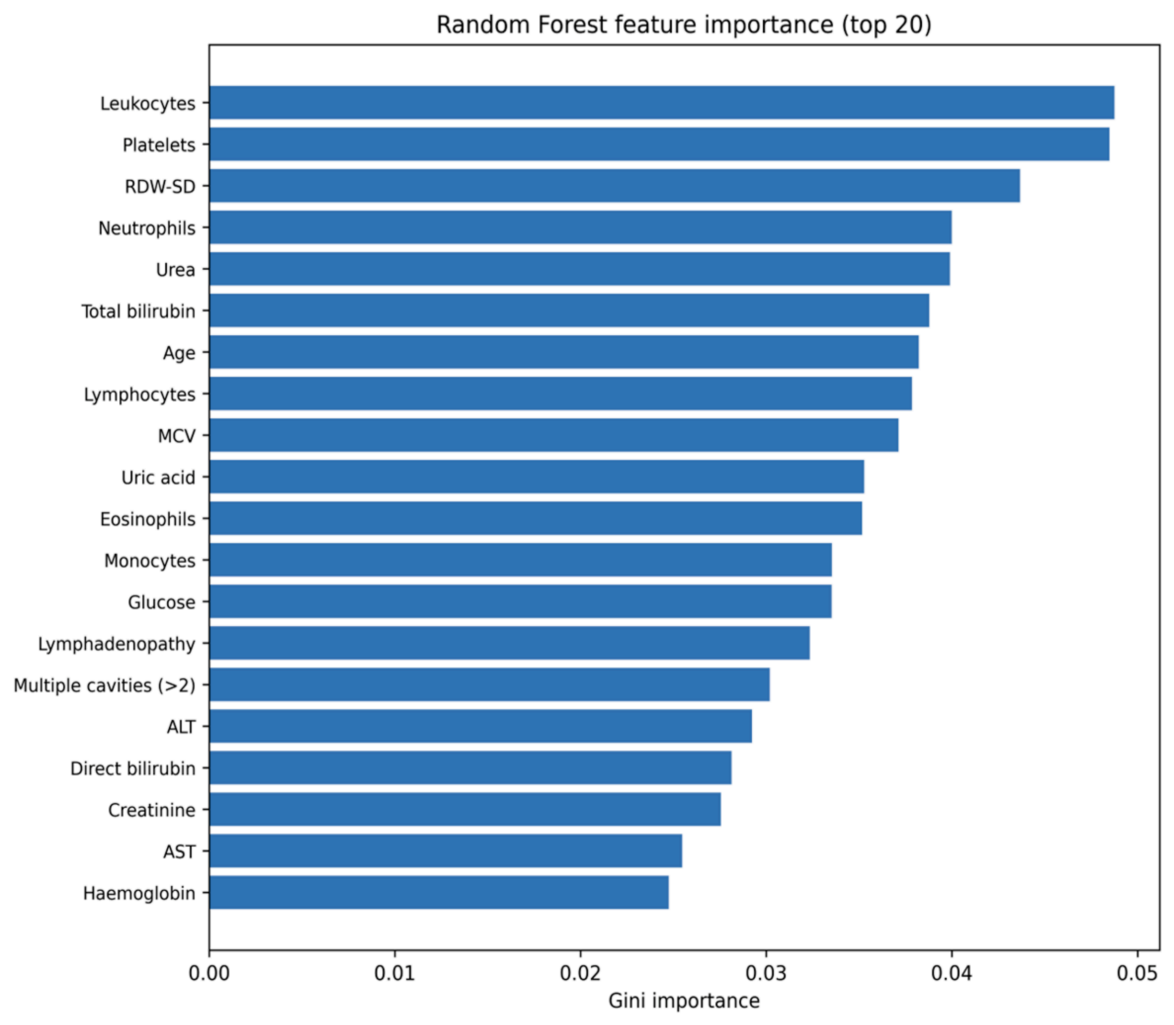
Random Forest feature importance (top 20 of all admission variables), shown as a descriptive all-variable screen. This analysis was separate from the compact candidate selection procedure; haematological inflammatory markers and cavity-related radiological features occupied leading positions in the Gini ranking.

To move from isolated associations toward a multivariable candidate set, a Random Forest was used as an exploratory screen. It achieved a held-out AUC of 0.63 and was therefore not used as the final prediction model. Its role was limited to descriptive ranking of potential predictors; leukocytes, platelets, RDW-SD, neutrophils and cavity/radiological features occupied leading positions, and held-out permutation importance was unstable, with several near-zero or negative estimates (Figure 2, Table 3). Because this screen was performed once in the full derivation cohort, its results should be interpreted as hypothesis-generating.

**Figure 3:**
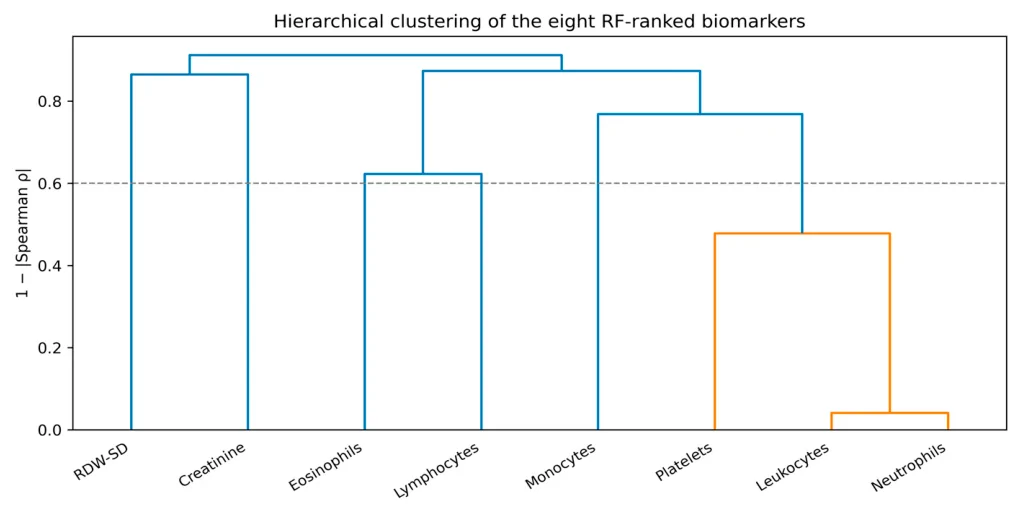
Hierarchical clustering of the eight Random-Forest-ranked continuous biomarkers (distance = 1 − |Spearman ρ|; dashed line = 0.6). The leukocyte–neutrophil–platelet cluster is represented by leukocytes; RDW-SD and monocytes were separate cluster representatives and, with leukocytes, had the three highest univariate AUCs among the representatives. Branch colours distinguish clusters below the 0.6 distance cut and have no additional quantitative meaning.

Within the defined continuous-biomarker pool, the eight highest-ranked variables were clustered by Spearman correlation distance (Figure 3). Leukocytes, neutrophils and platelets formed a correlated inflammatory burden cluster, with leukocytes retained as its representative because it had the highest univariate AUC. RDW-SD and monocytes were separate representatives; after representatives were ranked by univariate AUC, leukocytes, RDW-SD and monocytes were the top three and formed the fixed biomarker set. This full-cohort preselection is acknowledged in the interpretation of the conditional internal–validation results.

### 3.4. Construction of the Compact Score

**Figure 4:**
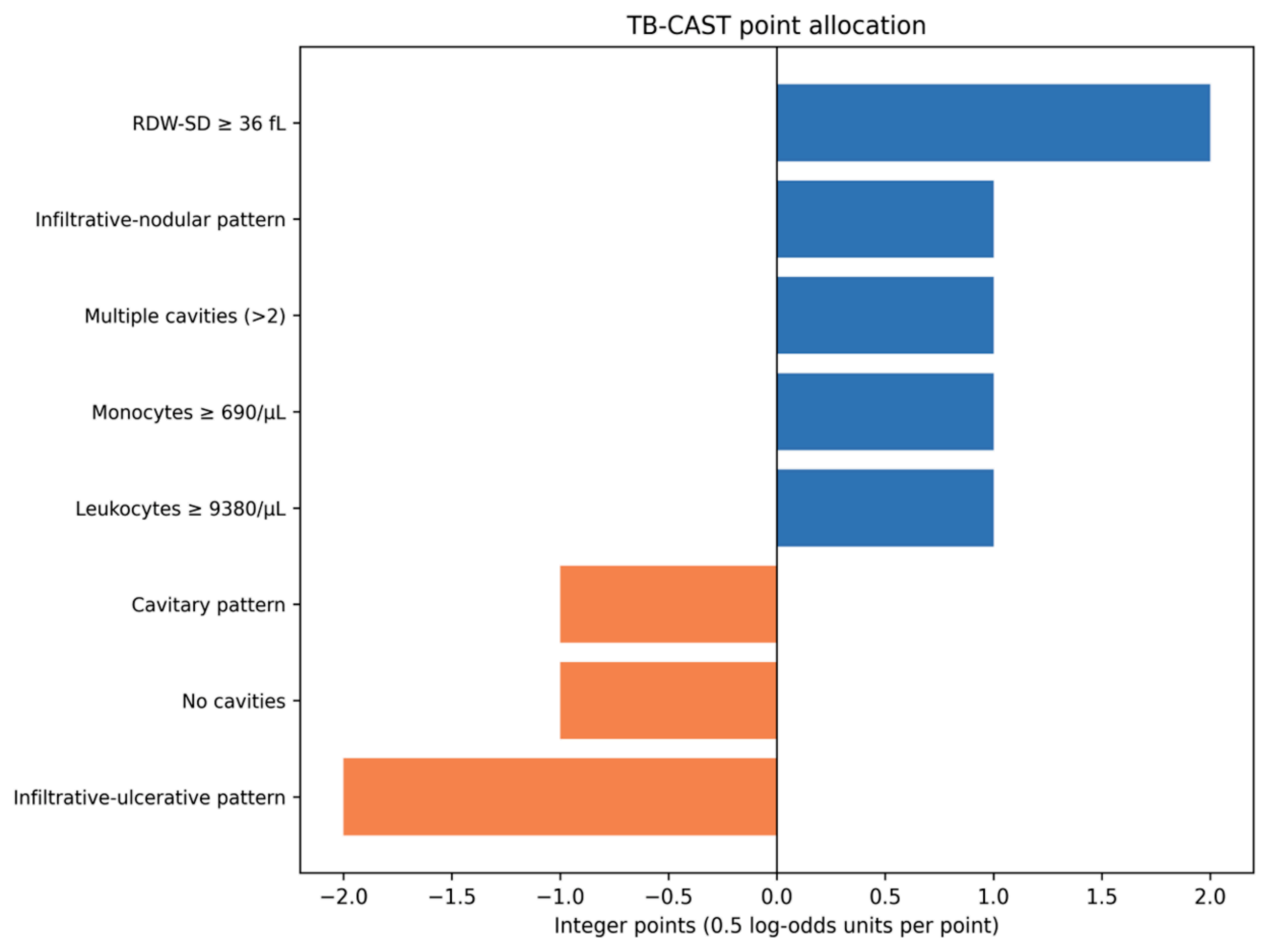
Integer points assigned in the full derivation model. Coefficients were divided by a fixed 0.5 log-odds unit and rounded; blue indicates risk-increasing and orange risk-decreasing components.

In the full derivation cohort, Youden cut-points were 9380/µL for leukocytes, 36 fL for RDW-SD and 690/µL for monocytes. Penalised logistic coefficients divided by the fixed 0.5 log-odds point unit produced the integer allocation shown in Table 4 and Figure 4. RDW-SD received +2 points; leukocytes, monocytes, multiple cavities and an infiltrative–nodular pattern received +1 point each; absence of cavities and a cavitary pattern each received −1 point; and an infiltrative–ulcerative pattern received −2 points. Radiological pattern points are expressed relative to the mixed pattern (code 4), which was the reference category. Total scores ranged from −3 to +5.

Full model specification (for reproducibility). The final L2-penalised logistic model (inverse regularisation C = 0.5, lbfgs solver, random seed 42) contained 12 fitted predictor terms plus the intercept (13 estimated parameters). The intercept was −1.8339. Coefficients and integer points were: leukocytes ≥9380/µL, +0.7184 (+1); RDW-SD ≥36 fL, +1.1142 (+2); monocytes ≥690/µL, +0.4470 (+1); one cavity, +0.1319 (0); two cavities, −0.2335 (0); >2 cavities, +0.6142 (+1), with no cavities as the cavity number reference; no cavities by cavity size, −0.5717 (−1); cavity <10 mm, +0.0559 (0); cavity 10–25 mm, +0.2115 (0), with >25 mm as the cavity size reference; infiltrative–nodular pattern, +0.3296 (+1); infiltrative–ulcerative pattern, −1.0303 (−2); and cavitary pattern, −0.6783 (−1), with mixed pattern as the radiological reference. Each fitted coefficient was divided by the fixed 0.5 log-odds point unit and rounded to the nearest integer. The total score maps to predicted probability as logit[P(positive)] = −2.4679 + 0.8291 × score. Across the observed score range, predicted probabilities were 0.007, 0.016, 0.036, 0.078, 0.163, 0.308, 0.505, 0.700 and 0.843 for scores −3 through +5, respectively. Supplementary Table S2 gives the complete fitted coefficient table and Supplementary Table S3 the full score-to-probability table. The complete analysis code, random-seed specification, exact resampling scheme and coefficient/risk tables are available from the corresponding author as stated in the Data Availability Statement.

**Figure 5:**
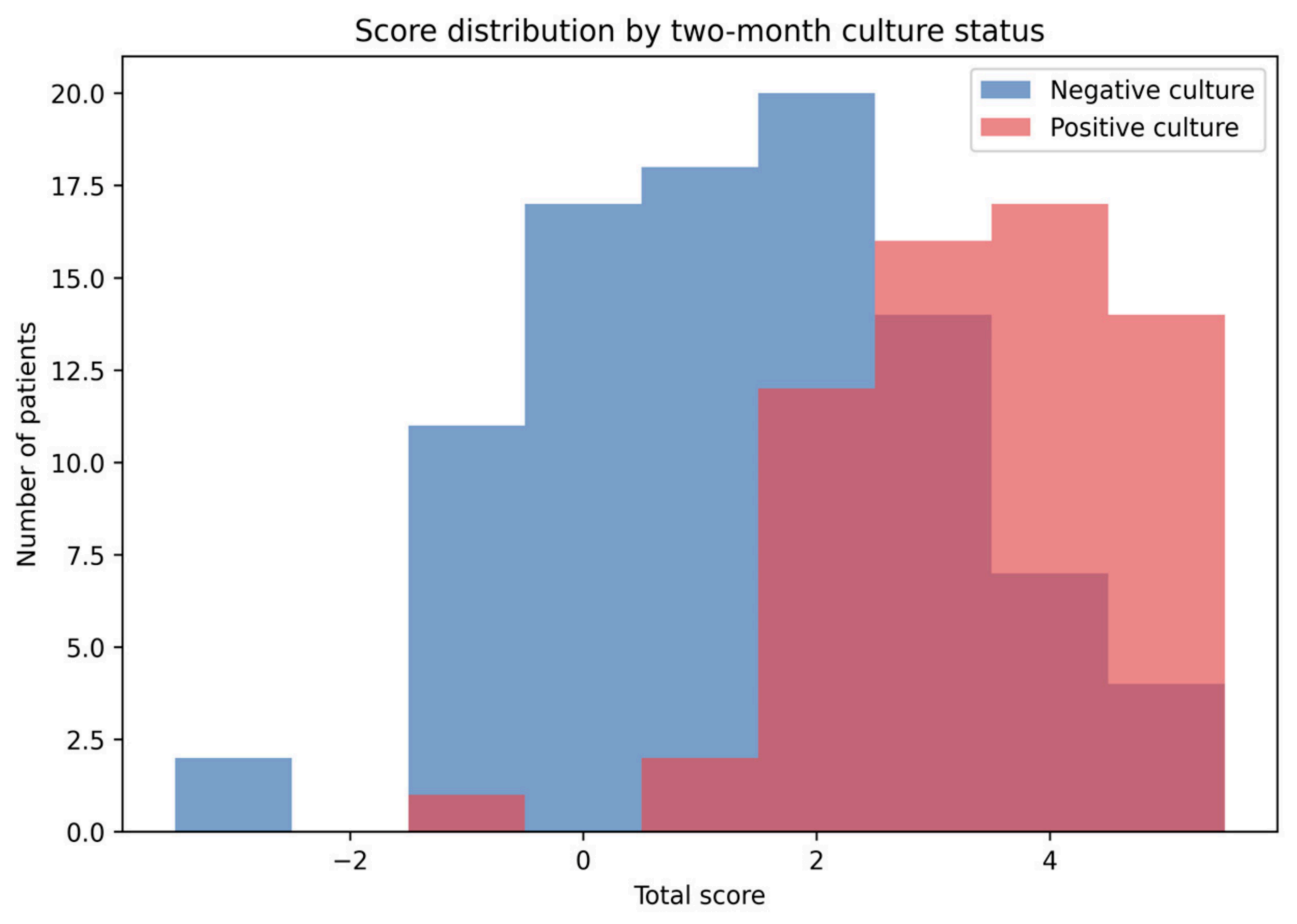
Distribution of the total score by two-month culture status. Culture-positive patients (red) are shifted toward higher scores, with a region of clinically informative overlap.

The resulting total score separated the two outcome groups: culture-positive patients were shifted toward higher values, and the two distributions, while overlapping, were clearly displaced (Figure 5). The overlap is expected for a baseline score and is precisely why a probabilistic, tuneable threshold—rather than a single hard cut-off—is appropriate (Section 3.6).

### 3.5. Univariate and Multivariable Analysis

The independent contribution of each dichotomised component was quantified by logistic regression, reported first as crude (univariate) odds ratios (Table 5) and then as mutually adjusted (multivariable) odds ratios (Table 6). In the univariate analysis, elevated leukocytes, RDW-SD, monocytes and multiple cavities were each significantly associated with culture positivity, whereas absence of cavities and the infiltrative–ulcerative pattern were associated with lower odds of culture positivity.

In the multivariable model (Table 6), which was highly significant overall (likelihood ratio *p* = 7.8 × 10^−9^; McFadden pseudo-R^2^ = 0.26), elevated RDW-SD emerged as the strongest independent predictor (adjusted OR 6.65, 95% CI 2.18–20.31), followed by elevated leukocytes (adjusted OR 2.43, 95% CI 1.08–5.48). The infiltrative–ulcerative pattern remained independently associated with lower odds relative to the mixed reference (adjusted OR 0.12, 95% CI 0.02–0.62). Monocytes and multiple cavities lost significance after adjustment. These adjusted estimates are exploratory: in a cohort of 155 patients, they may be unstable, and they should be read as associations within this derivation sample rather than as confirmation of predictor effects.

### 3.6. Cross-Validated Performance and Operating Points

Fully nested validation. To evaluate the complete data-driven development process, Random Forest ranking, correlation clustering, representative selection, selection of the three highest-AUC representatives, cut-point estimation, penalised regression, fixed-scale integer-point conversion and score-to-probability mapping were repeated inside every training fold and evaluated only on the corresponding untouched test fold. This fully nested procedure gave a mean test-fold AUC of 0.70 ± 0.09 (fold-level percentile interval 0.53–0.85) and is the primary internal estimate for the complete pipeline. Leukocytes were selected in 94% of folds, RDW-SD in 70% and monocytes in 40%; the lower monocyte frequency and selection of alternative biomarkers in some folds demonstrate material selection instability.

**Figure 6:**
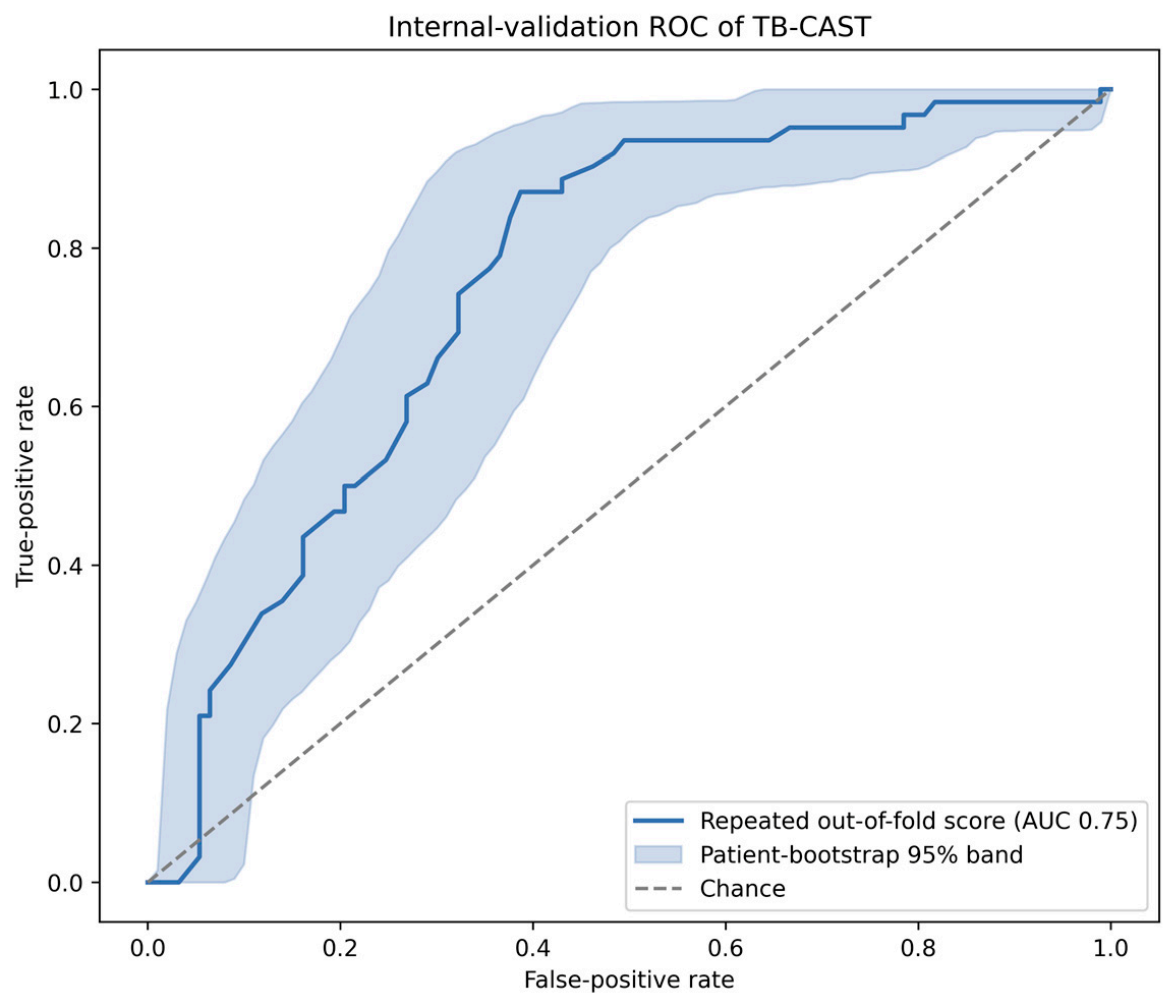
ROC curve of the patient-level mean repeated out-of-fold TB-CAST score from the conditional analysis with the full-cohort-selected candidate set held fixed. AUC = 0.75; shaded area is the patient-level bootstrap 95% band (AUC 95% CI 0.67–0.83).

Conditional analysis. With the full-cohort-selected candidate set held fixed, 10 repeated stratified 5-fold partitions gave a mean test-fold AUC of 0.74 ± 0.08. When the 10 out-of-fold scores for each patient were averaged, patient-level discrimination was 0.75 (bootstrap 95% CI 0.67–0.83; Figure 6). These two estimates are conditional on full-cohort predictor preselection and should not be interpreted as validation of the complete selection process. Fold-wise cut-points also varied: median (interquartile range) values were 9050 (7280–9380)/µL for leukocytes, 35.7 (35.7–35.8) fL for RDW-SD and 690 (615–690)/µL for monocytes.

Stability of the fixed-point system. With the candidate set held fixed at the published components and the 0.5 log-odds point scale, the assigned point matched the full-cohort value in 96% of folds for absence of cavities, 92% for the infiltrative–ulcerative pattern, 92% for the cavitary pattern, 88% for multiple cavities, 78% for RDW-SD, 78% for monocytes and 72% for the infiltrative–nodular pattern. Leukocyte points were notably less stable: the full-cohort value of +1 occurred in 20% of folds, while +2 occurred most often. RDW-SD and monocyte cut-points were relatively stable (36 fL and 690/µL in most folds), whereas the leukocyte cut-point ranged from 7280 to 9720/µL. The published TB-CAST points should therefore be read as a representative full-cohort specification rather than as a uniquely determined solution.

**Figure 7:**
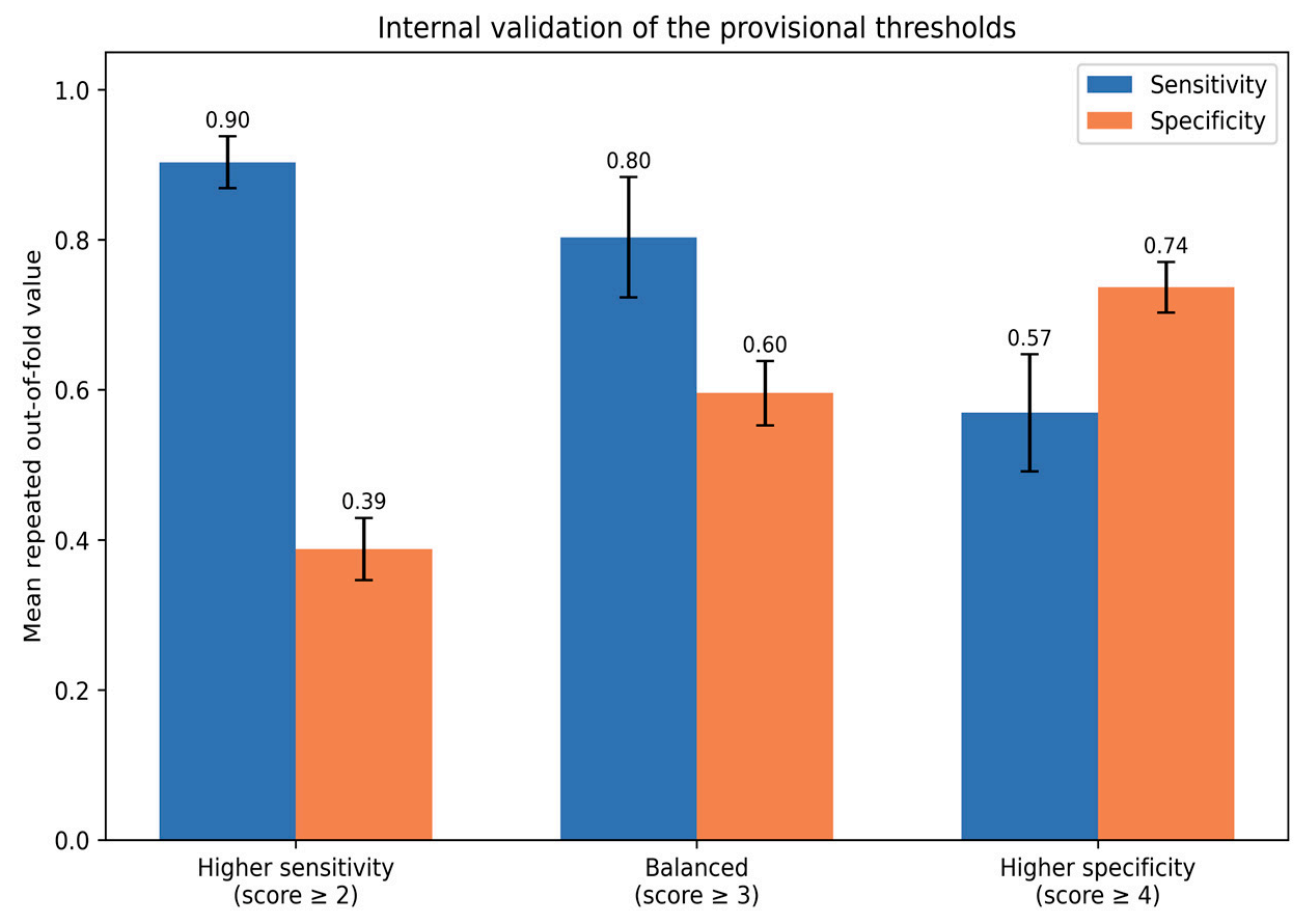
Mean sensitivity and specificity of the three provisional thresholds in the conditional procedure across 10 repeated stratified 5-fold partitions; error bars show the standard deviation across repetitions. The estimates reflect fold-specific re-estimation and do not directly validate the exact fixed Table 4 score.

The final derivation model had an apparent AUC of 0.825; bootstrap correction reduced this to 0.786 (estimated optimism 0.039). This secondary correction does not account for the initial full-cohort candidate preselection and should not replace the fully nested complete-pipeline estimate. At fixed thresholds on the common 0.5-log-odds point scale, the conditional modelling procedure gave mean sensitivity/specificity of 0.90 ± 0.03/0.39 ± 0.04 for ≥2 points, 0.80 ± 0.08/0.60 ± 0.04 for ≥3 points, and 0.57 ± 0.08/0.74 ± 0.03 for ≥4 points (Table 7 and Figure 7). Within each partition, cut-points, coefficients, integer points and the probability mapping were re-estimated from the training data. These threshold-specific estimates therefore evaluate the modelling procedure conditional on the fixed candidate set; they do not directly validate the exact fixed full-cohort score shown in Table 4 and the illustrative scorecard in Section 4.5.

### 3.7. Calibration, Clinical Utility and Explainability

**Figure 8:**
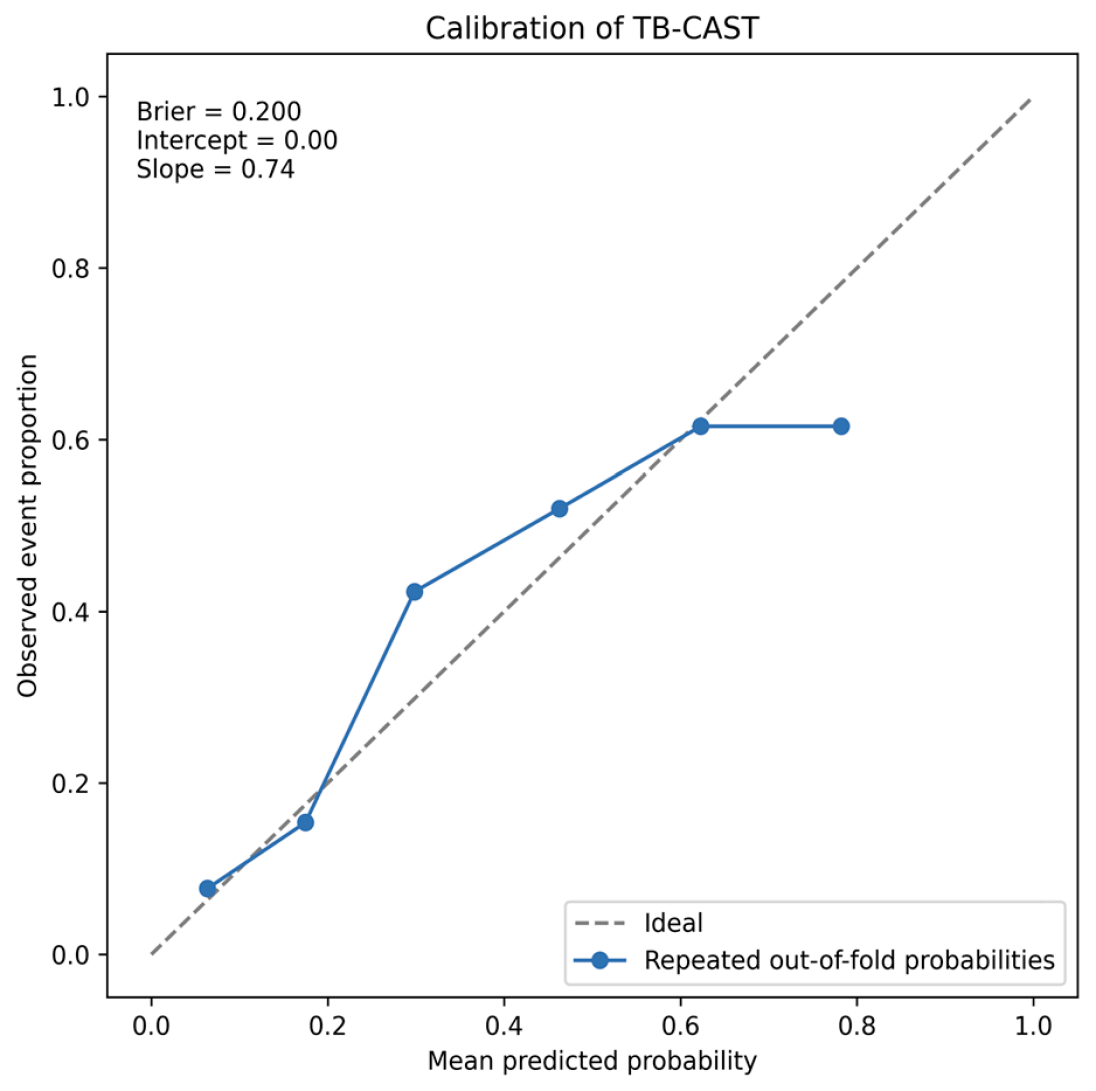
Calibration of averaged repeated out-of-fold probabilities from the conditional analysis with the full-cohort-selected candidate set held fixed. Brier score = 0.200, calibration intercept = −0.00 and calibration slope = 0.74.

**Figure 9:**
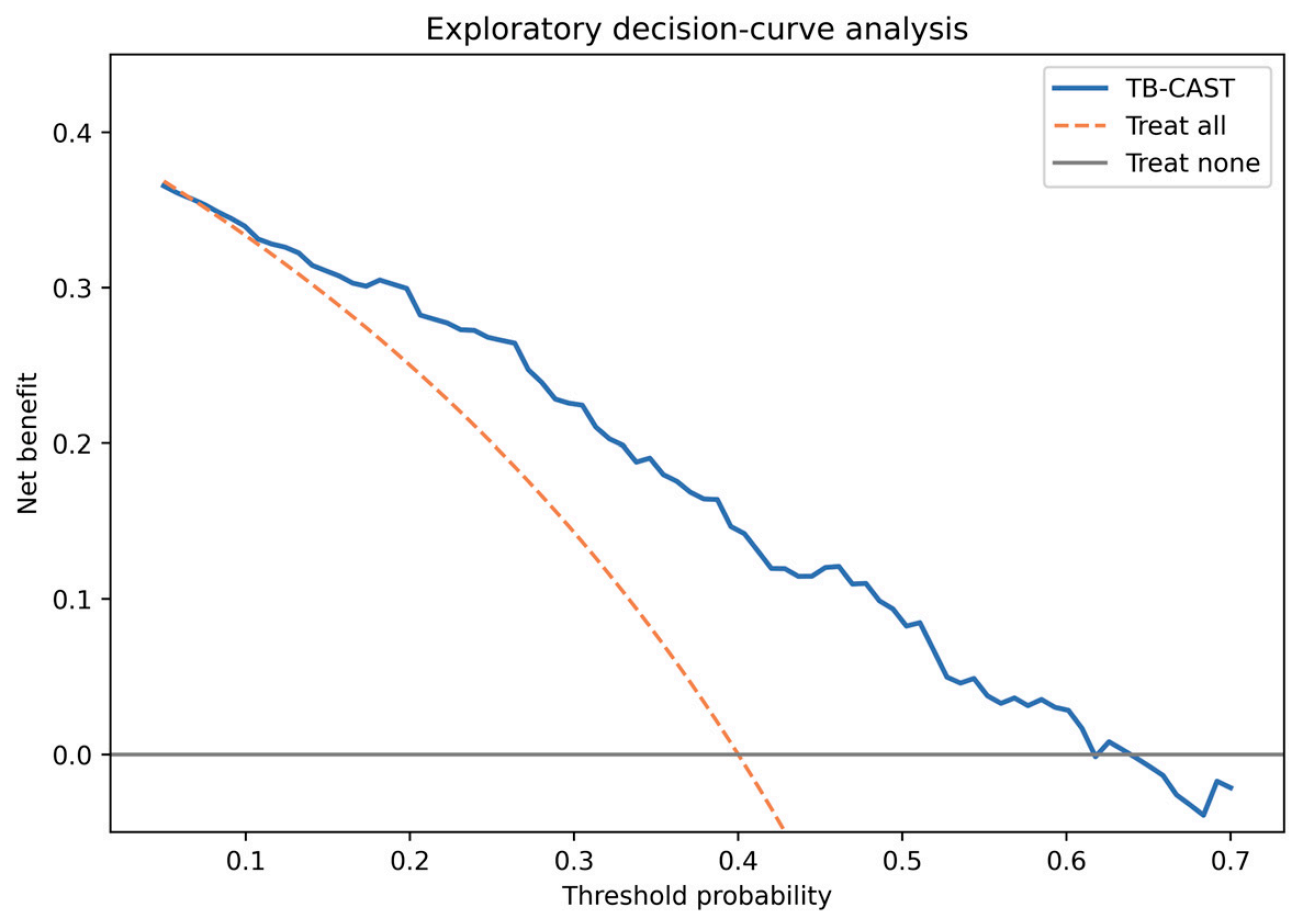
Exploratory decision curve analysis based on averaged repeated out-of-fold probabilities. Net benefit estimates require external validation and should not be interpreted as evidence of clinical effectiveness.

Conditional on the full-cohort-selected candidate set, the average repeated out-of-fold probabilities gave a Brier score of 0.200 (patient bootstrap 95% CI 0.166–0.237), a calibration intercept of −0.00 (95% CI −0.40 to 0.40), and a calibration slope of 0.74 (95% CI 0.45–1.12) (Figure 8). The slope below 1 suggests some overfitting, although its confidence interval included 1. Decision curve analysis was exploratory and indicated potential net benefit over selected threshold probabilities (Figure 9); it should not be interpreted as evidence of clinical effectiveness.

**Figure 10:**
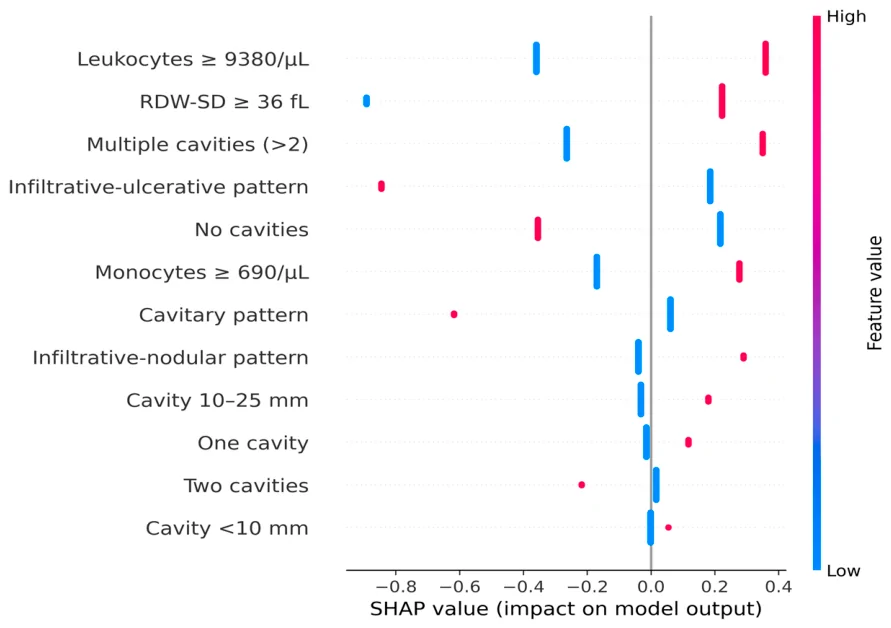
Post hoc SHAP summary of the final derivation model; features are ranked by mean absolute contribution. This is a descriptive explanation, not an external validation analysis.

**Figure 11:**
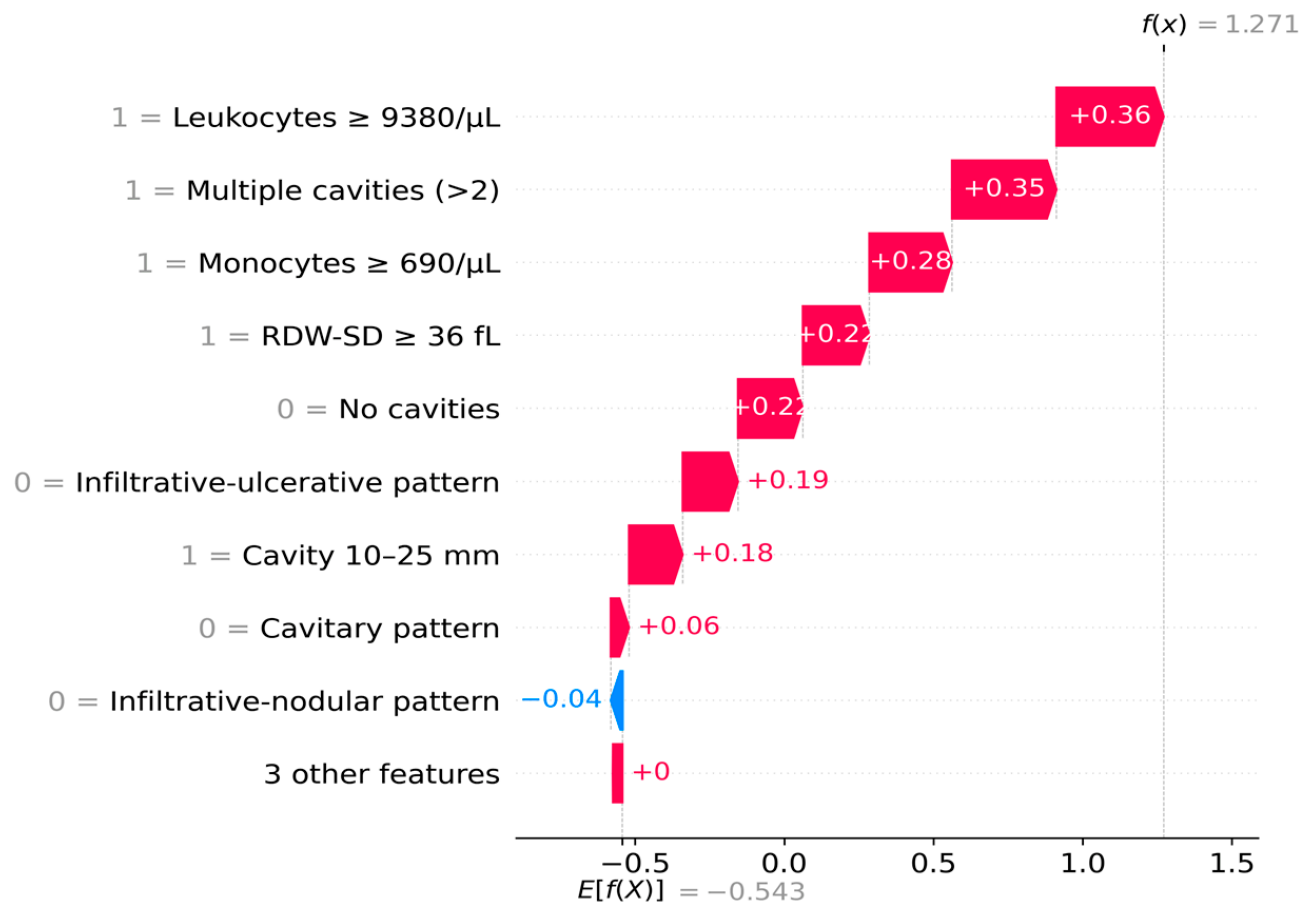
Post hoc SHAP waterfall for a representative high-risk derivation-cohort patient, showing additive contributions to the fitted log-odds. Red contributions increase the model output relative to the baseline value, whereas blue contributions decrease it.

**Figure 12:**
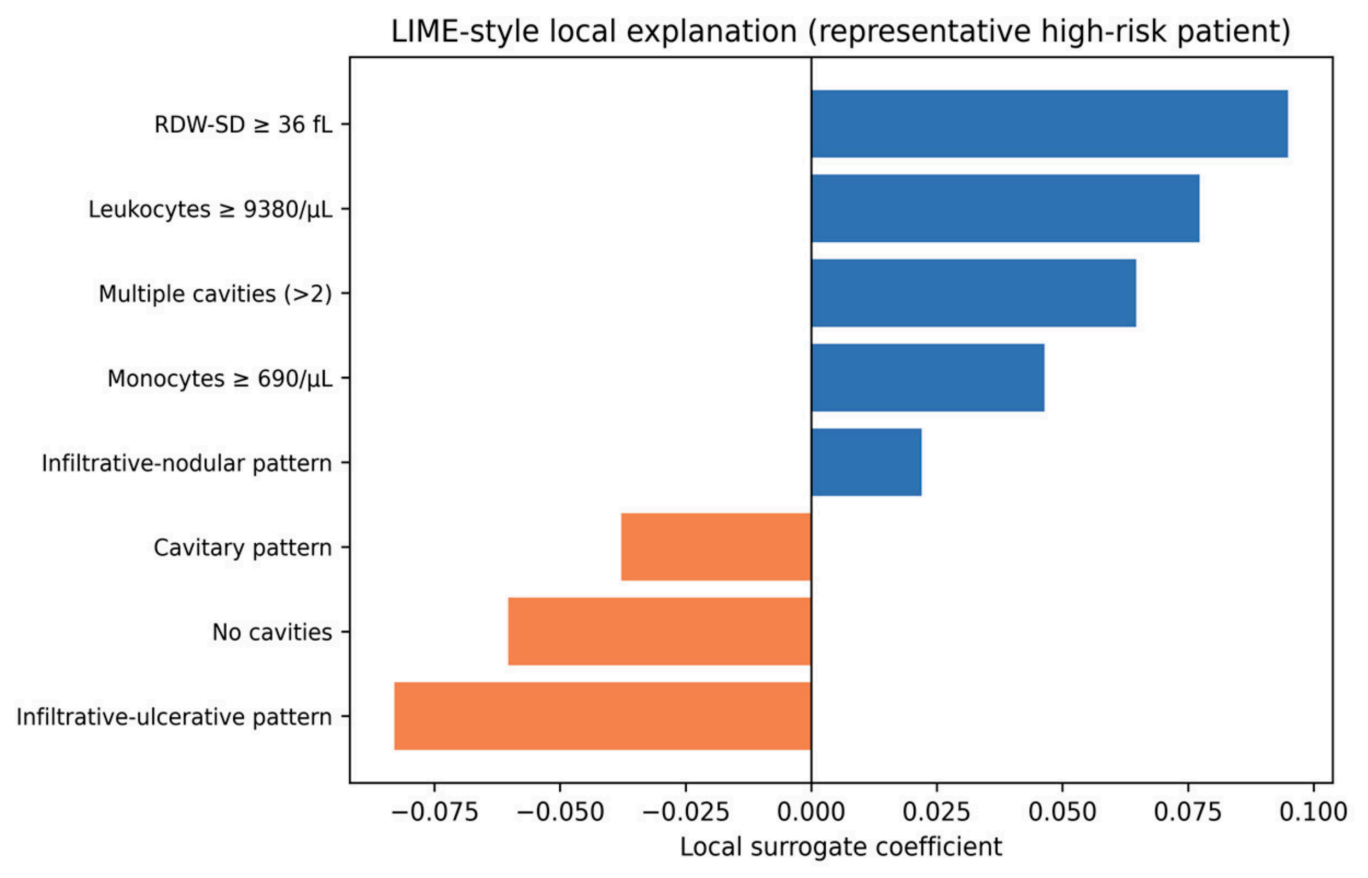
LIME-style local weighted-surrogate explanation for the same derivation-cohort patient. The local ranking is descriptive and does not establish transportability.

Post hoc SHAP and a LIME-style local weighted surrogate were used to describe the fitted derivation model rather than to validate it. SHAP assigned the largest absolute contributions to leukocytes, RDW-SD and cavity-related features (Figure 10); these values explain the pre-rounded penalised model, so the cavity and pattern indicators displayed include categories that round to zero points and therefore do not all appear among the non-zero components in Table 4. The waterfall plot for a representative high-risk patient decomposed the fitted log-odds into additive feature contributions (Figure 11), and the local surrogate provided a similar patient-level ordering (Figure 12). These descriptive explanations do not demonstrate transportability or causal relevance.

### 3.8. Provisional Operating Thresholds and Worked Examples

**Figure 13:**
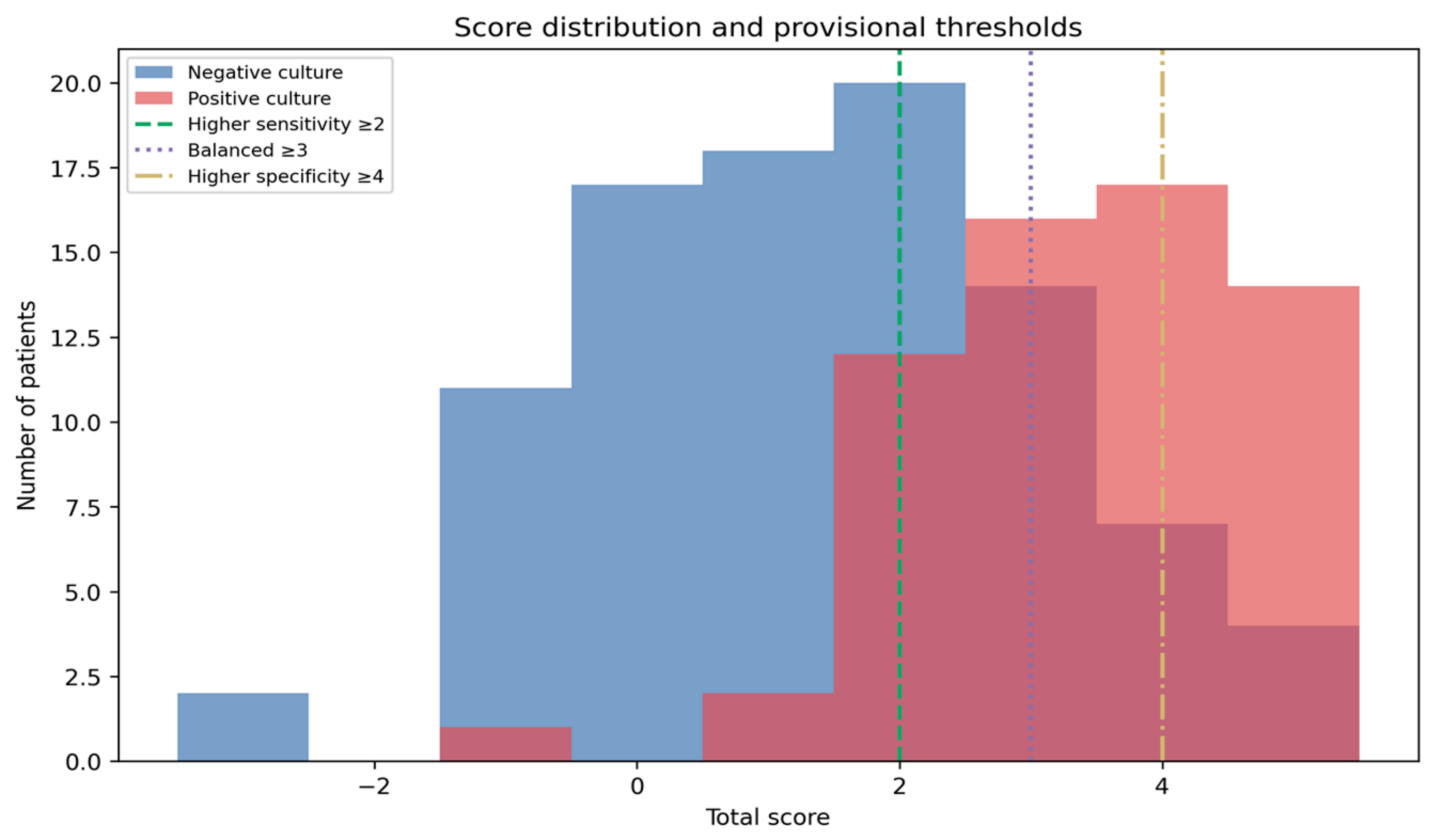
Distribution of the fixed full-cohort TB-CAST score by two-month culture status, with the three provisional thresholds (higher-sensitivity ≥2, balanced ≥3, higher-specificity ≥4). Shifting the threshold rightward trades sensitivity for specificity. Threshold operating characteristics reported in Table 7 and Table 8 come from the conditional fold-specific procedure and do not directly validate this exact fixed score.

Because the acceptable balance between sensitivity and specificity depends on the intended action, we present three provisional thresholds applied to the TB-CAST point scale (Table 8, Figure 13). In the conditional repeated-validation procedure, the ≥2-point threshold prioritised sensitivity (0.90) but had low specificity (0.39); the ≥3-point threshold provided a more balanced profile (sensitivity 0.80, specificity 0.60); and the ≥4-point threshold increased specificity to 0.74 while sensitivity fell to 0.57. These estimates were obtained after fold-specific re-estimation of cut-points, coefficients, points and probability mappings and therefore describe the conditional modelling procedure rather than direct validation of the exact fixed full-cohort score. Apparent derivation-cohort predictive values and odds ratios are shown separately and should not be used as validated operating characteristics. The thresholds are intended to illustrate trade-offs for future validation, not to prescribe centre-level clinical policy.

Table 9 shows three worked derivation-cohort examples. A patient with elevated leukocytes, RDW-SD and monocytes plus multiple cavities scores +5 and exceeds all three thresholds; a patient with a total of +3, including a cavitary pattern contribution of −1, exceeds the ≥2 and ≥3 thresholds but not ≥4; and a patient with no cavities and an infiltrative–ulcerative pattern scores −3 and remains below all thresholds. These examples demonstrate arithmetic application of the score only and should not be interpreted as prospective clinical validation.

## 4. Discussion

### 4.1. Main Findings

Using baseline data available before anti-TB therapy, we derived a compact score based on leukocytes, RDW-SD, monocytes and cavity/radiological features. The fully nested analysis, which repeated the entire data-driven pipeline within each training fold, yielded a mean test-fold AUC of 0.70 ± 0.09 and is the most appropriate internal estimate for the complete development procedure. Secondary analyses conditional on the full-cohort-selected candidate set yielded a mean test-fold AUC of 0.74 ± 0.08 and a patient-level mean repeated out-of-fold score AUC of 0.75 (95% CI 0.67–0.83). Apparent derivation performance (AUC 0.825) was higher. The gap between fully nested and conditional estimates, together with variability in selected biomarkers, cut-points and integer weights and a conditional calibration slope of 0.74, indicates selection instability and potential optimism. TB-CAST should therefore be viewed as a candidate score requiring external validation and, if necessary, recalibration rather than as a clinically validated tool.

### 4.2. Biological Plausibility

The retained predictors are mechanistically coherent. Elevated admission leukocyte and neutrophil counts are repeatedly associated with more severe TB, greater dissemination and slower culture conversion, as neutrophils release proinflammatory mediators that promote tissue damage and cavitation [27,28,29]. RDW-SD, which emerged here as the strongest independent predictor, reflects anisocytosis from chronic inflammation and impaired erythropoiesis and has been linked to anaemia of inflammation and to disease severity in TB [30,31]. Monocytes are central to antimycobacterial immunity, and elevated counts or specific subsets are seen in more resistant, prolonged disease [32,33]. Pulmonary cavity number and size are established prognostic factors reflecting high bacillary load and impaired drug penetration [34,35]. The negative points attached to some radiological patterns reflect dataset-specific associations and should be interpreted cautiously; no single imaging finding provides a complete risk picture, and these categories must be read within the multiparametric framework.

### 4.3. Comparison with Existing Scores

For the complete data-driven TB-CAST pipeline, the fully nested mean test-fold AUC of 0.70 ± 0.09 is the most appropriate internal performance estimate (Table 10). Conditional analyses using the candidate set selected once in the full cohort yielded a mean fold AUC of 0.74 ± 0.08 and a patient-level mean repeated out-of-fold score AUC of 0.75; these values are retained as secondary conditional estimates rather than as validation of the complete development process. A clinical indicator score using ten haematological indices plus cavity and smoking status reported AUCs of 0.77 in derivation and 0.80 in external validation [36]. A three-item MDR-TB outcome score reported AUCs of 0.67–0.69 [37], and a nomogram for unsuccessful MDR-PTB outcomes reached 0.76 [38]. Multimodal models incorporating microbiological and genomic information have reported higher performance; Sambarey et al. combined 203 clinical, radiological, microbiological and pathogen-genomic features in 5060 patients and reported an AUC of 0.84 [39]. A companion study from our group reported an out-of-fold AUC of 0.88 for a different endpoint—mortality in TB with severe malnutrition [25]. These comparisons are contextual rather than head-to-head because outcomes, populations and validation designs differ.

### 4.4. Interpretation at Different Thresholds: A Proposed, Not Yet Validated, Feature

A practical feature of TB-CAST is that a single point total can be interpreted at different provisional thresholds. This makes the sensitivity–specificity trade-off explicit rather than implying that one cut-off is universally optimal. In the conditional repeated-validation procedure, threshold performance differed materially from apparent full-cohort values, particularly for the higher-specificity option. These thresholds should therefore be regarded as candidates for prospective evaluation and local recalibration, not as ready-to-use centre policies. Because no centre-level recalibration or cross-institutional evaluation was performed, centre adaptation remains a proposed future use rather than an empirically demonstrated property of the score.

The ≥2 threshold may be studied as a higher-sensitivity option, the ≥3 threshold as a balanced option, and the ≥4 threshold as a higher-specificity option. In the conditional fold-specific modelling procedure, their mean sensitivity/specificity were 0.90/0.39, 0.80/0.60 and 0.57/0.74, respectively. These values do not directly validate the exact fixed full-cohort score and do not support claims that a low score can safely de-prioritise care or that a high score can independently allocate scarce treatment resources. Any future use should remain adjunctive to guideline-concordant assessment, and threshold selection should be based on external validation, local prevalence, the consequences of false results and a prespecified clinical action.

### 4.5. Implementation as a Printed Form or Mobile Application

**Figure 14:**
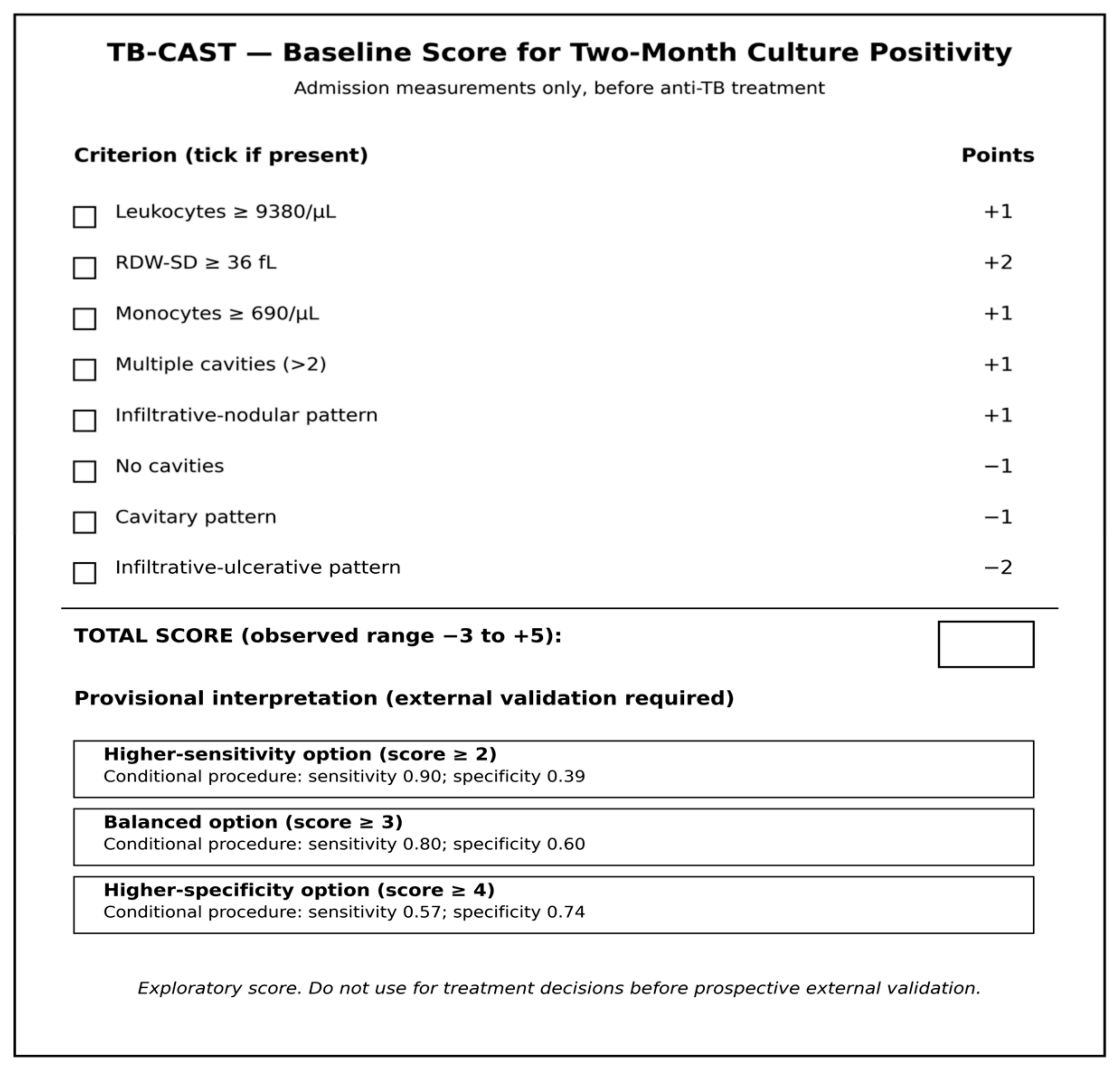
Illustrative TB-CAST bedside form showing the eight non-zero components of the fixed full-cohort score and three provisional thresholds. The displayed threshold operating characteristics are conditional procedure estimates obtained after fold-specific re-estimation and do not directly validate this exact fixed scorecard. Prospective external validation is required before clinical use.

TB-CAST was designed as a short additive score that can be calculated on paper or in software. Figure 14 presents an illustrative bedside form with the eight non-zero components and the three provisional thresholds. A future digital implementation could automate arithmetic, record the selected threshold and display uncertainty, but should not provide treatment recommendations until prospective external validation and impact assessment are complete. The current figures describe a design concept rather than a deployed medical device.

After independent external validation, transparent model documentation and appropriate governance, TB-CAST could be considered for implementation as an online calculator. At the present stage, sharing the analytic code, variable definitions and reporting aligned with TRIPOD principles [40] is more important than immediate deployment. The score is intended only to support prioritisation of standard monitoring and must not replace guideline-concordant diagnosis, treatment or drug susceptibility assessment.

### 4.6. Limitations

This study has several limitations. It is a single-centre derivation study with 155 patients and 62 events, and no a priori sample size calculation was performed. The fully nested analysis provides the primary internal estimate for the complete data-driven pipeline but remains an internal validation; the secondary AUCs of 0.74 and 0.75 and the threshold-specific operating characteristics are conditional on full-cohort predictor preselection and may retain selection optimism. Biomarker selection, cut-points and several integer weights varied across training folds, and the three thresholds remain provisional. The conditional calibration slope was below 1, consistent with some overfitting. External validation in independent cohorts is required before clinical or digital implementation. Only treatment-naïve, HIV-negative patients able to provide valid sputum were included, limiting generalisability. The archived data do not retain an imaging modality indicator or separate CT/radiograph ratings, so modality-specific counts and formal modality harmonisation cannot be reconstructed. Two source-coded cavity number/cavity size discordances were identified; a sensitivity harmonisation produced no change in rounded score components and only negligible changes in conditional performance, but the original imaging could not be adjudicated from the locked analytic files. The dataset also does not retain a validated rifampicin resistance or phenotypic drug susceptibility classification, individual treatment regimens or adherence, so MDR/RR/XDR distribution and resistance- or regimen-specific effects cannot be reported reliably. Baseline bacillary-load measures such as smear grade or Xpert cycle threshold and pathogen lineage were not modelled. Complete baseline data were an inclusion requirement, which avoids imputation but may reduce applicability where testing is incomplete. Finally, SHAP, the LIME-style local surrogate and decision curve analyses were post hoc descriptive analyses and do not establish causal effects or clinical impact.

## 5. Conclusions

We derived TB-CAST, a parsimonious baseline score for two-month sputum culture positivity using routine laboratory and radiological variables measured before treatment. The fully nested complete-pipeline analysis yielded a mean test-fold AUC of 0.70 ± 0.09 and should be regarded as the primary internal performance estimate. Conditional on the candidate set selected once in the full cohort, mean test-fold AUC was 0.74 ± 0.08, and patient-level repeated out-of-fold score AUC was 0.75 (95% CI 0.67–0.83); the conditional Brier score was 0.200 and calibration slope 0.74. The ≥2, ≥3 and ≥4 threshold estimates likewise describe the conditional fold-specific modelling procedure rather than direct validation of the exact fixed full-cohort score. These thresholds remain provisional. Prospective multicentre external validation, calibration assessment and clinical impact evaluation are required before implementation.

## Figures and Tables

**Table 1:** Baseline demographic and continuous laboratory parameters by two-month culture status (admission values; mean ± SD for Welch-tested variables, median [IQR] for Mann–Whitney U variables, and *n* [%] for categorical variables).

Parameter	Positive (*n* = 62)	Negative (*n* = 93)	Test	*p*
Age (years)	52.1 ± 12.4	50.8 ± 13.6	*t*-test	0.537
Male sex, *n* (%)	49 (79.0)	73 (78.5)	Fisher	1.000
BMI (kg/m^2^)	19.1 [17.0–21.2]	19.4 [17.9–22.4]	MWU	0.165
Leukocytes (/µL)	10,390 [8638–11,865]	8400 [6490–11,030]	MWU	0.001 *
Neutrophils (/µL)	7450 [5713–9360]	6160 [4380–7950]	MWU	0.003 *
Platelets (/µL)	326,500 [272,500–420,500]	283,000 [227,000–352,000]	MWU	0.036 *
Monocytes (/µL)	615 [355–840]	500 [320–720]	MWU	0.184
RDW-SD (fL)	41.3 [37.5–44.4]	40.4 [34.9–44.0]	MWU	0.109
Eosinophils (/µL)	135 [60–285]	110 [40–190]	MWU	0.205
Uric acid (mg/dL)	4.3 [3.5–5.3]	4.5 [3.8–5.7]	MWU	0.412

MWU = Mann–Whitney U test; IQR = interquartile range; RDW-SD = red-cell distribution width–standard deviation; * *p* < 0.05.

**Table 2:** Clinical and imaging parameters by two-month culture status (baseline thoracic imaging).

Clinical/Imaging Parameter	Test	Effect/Test Statistic	*p*
Radiological pattern	Chi-square	17.64	0.0005 *
Number of cavities	Chi-square	17.13	0.0007 *
Lymphadenopathy	Fisher	3.86	0.0015 *
Cavity size	Chi-square	13.72	0.0033 *
Extent of lung involvement	Chi-square	15.41	0.0087 *
History of tumours	Fisher	0.14	0.051
Dry cough	Fisher	0.37	0.051
Productive cough	Fisher	2.34	0.068

* *p* < 0.05. For 2 × 2 comparisons, odds ratios are conditional maximum-likelihood estimates for category 1/present versus category 2/absent, with exact 95% CIs: lymphadenopathy OR 3.86 (1.58–10.52); tumours OR 0.14 (0.003–1.01); dry cough OR 0.37 (0.11–1.03); and productive cough OR 2.34 (0.92–6.51). For multi-category comparisons: extent of involvement χ^2^ = 15.41, df = 5; cavity number χ^2^ = 17.13, df = 3; cavity size χ^2^ = 13.72, df = 3; and radiological pattern χ^2^ = 17.64, df = 3.

**Table 3:** Selected Gini and held-out permutation importance estimates from the exploratory Random Forest; permutation estimates were unstable and were not used as the sole basis for retention.

Predictor	Gini Importance	Permutation Importance
Leukocytes	0.049	0.034
Platelets	0.048	−0.013
RDW-SD	0.044	≈0.000
Neutrophils	0.040	≈0.000
Monocytes	0.034	0.010
Lymphadenopathy	0.032	0.015
Multiple cavities (>2)	0.030	0.021
Cavity size (all indicators)	0.028	0.017
Infiltrative–ulcerative pattern	0.021	0.017

**Table 4:** Components and integer points of the compact data-driven score (baseline admission values).

Component	Definition	Points
RDW-SD	≥36 fL	+2
Leukocytes	≥9380/µL	+1
Monocytes	≥690/µL	+1
Number of cavities	multiple (>2)	+1
Radiological pattern	infiltrative–nodular	+1
Cavity size	none	−1
Radiological pattern	cavitary	−1
Radiological pattern	infiltrative–ulcerative	−2

*Categorical points are expressed relative to the fixed reference categories: no cavities for cavity number, >25 mm for cavity size, and mixed for radiological pattern. Components not listed contributed 0 points.*

**Table 5:** Univariate (crude) odds ratios for the score components (admission values).

Component	Crude OR (95% CI)	*p*
Leukocytes ≥ 9380/µL	3.43 (1.73–6.77)	<0.001
RDW-SD ≥ 36 fL	4.19 (1.51–11.65)	0.004
Monocytes ≥ 690/µL	2.15 (1.10–4.20)	0.028
Multiple cavities (>2)	3.57 (1.81–7.03)	<0.001
Infiltrative–nodular pattern	1.38 (0.50–3.80)	0.603
No cavities	0.26 (0.12–0.54)	<0.001
Cavitary pattern	0.31 (0.06–1.49)	0.201
Infiltrative–ulcerative pattern	0.10 (0.02–0.42)	<0.001

*OR = odds ratio; CI = confidence interval.*

**Table 6:** Multivariable logistic regression: adjusted odds ratios for the score components.

Component	Adjusted OR (95% CI)	*p*
RDW-SD ≥ 36 fL	6.65 (2.18–20.31)	0.001
Leukocytes ≥ 9380/µL	2.43 (1.08–5.48)	0.033
Multiple cavities (>2)	2.05 (0.77–5.44)	0.151
Monocytes ≥ 690/µL	1.66 (0.73–3.78)	0.230
Infiltrative–nodular pattern	1.58 (0.45–5.50)	0.476
No cavities	0.47 (0.15–1.45)	0.190
Cavitary pattern	0.19 (0.04–1.03)	0.054
Infiltrative–ulcerative pattern	0.12 (0.02–0.62)	0.012

*Adjusted ORs are mutually adjusted for all listed components. Model: pseudo-R^2^ = 0.26, likelihood ratio p = 7.8 × 10^−9^.*

**Table 7:** Conditional-procedure operating characteristics. Values are means across 10 repeated stratified 5-fold partitions; ± values are standard deviations across repetitions. Within each partition, cut-points, coefficients, integer points and probability mapping were re-estimated using training data only.

Operating Point	Sensitivity	Specificity	PPV	NPV
Higher-sensitivity option (score ≥ 2)	0.90 ± 0.03	0.39 ± 0.04	0.50 ± 0.02	0.86 ± 0.04
Balanced (score ≥ 3)	0.80 ± 0.08	0.60 ± 0.04	0.57 ± 0.03	0.82 ± 0.05
Higher-specificity option (score ≥ 4)	0.57 ± 0.08	0.74 ± 0.03	0.59 ± 0.03	0.72 ± 0.03
Fold-specific Youden	0.81	0.60	0.58	0.84

PPV = positive predictive value; NPV = negative predictive value. These estimates are conditional on the full-cohort-selected candidate predictor set and evaluate the fold-specific modelling procedure, not the exact fixed full-cohort score in Table 4.

**Table 8:** Three provisional TB-CAST thresholds: conditional-procedure sensitivity/specificity and apparent derivation-cohort predictive values. The conditional estimates were obtained after fold-specific re-estimation and do not directly validate the exact fixed full-cohort score.

Mode	Internal Se	Internal Sp	Deriv. PPV	Deriv. NPV	Deriv. OR (95% CI)
Higher-sensitivity option (score ≥ 2)	0.90	0.39	0.57	0.94	21.0 (6.1–71.7)
Balanced (score ≥ 3)	0.80	0.60	0.65	0.82	8.5 (4.1–17.9)
Higher-specificity option (score ≥ 4)	0.57	0.74	0.74	0.73	7.5 (3.3–16.6)

PPV = positive predictive value; NPV = negative predictive value; OR = odds ratio. Sensitivity and specificity are means from the 10 repeated 5-fold conditional procedure. PPV, NPV and OR are apparent full-derivation-cohort values and are reported only for descriptive comparison.

**Table 9:** Worked examples: computing TB-CAST for three derivation-cohort patients.

Patient (Baseline Values)	Points Awarded	Total	≥2	≥3	≥4
LEU 9420; RDW-SD 51 fL; MONO 690; >2 cavities	+1, +2, +1, +1	+5	Above	Above	Above
LEU 10,410; RDW-SD 37 fL; MONO 780; cavitary pattern	+1, +2, +1, −1	+3	Above	Above	Below
No cavities; infiltrative–ulcerative pattern (LEU 7160; RDW-SD 35 fL; MONO 460)	−1, −2	−3	Below	Below	Below

“Above” denotes a score at or above the selected provisional threshold, and “Below” denotes a score below it. The examples are from the derivation cohort and are included to demonstrate calculation, not to estimate prospective performance.

**Table 10:** Comparison of TB-CAST with selected published TB prediction models.

Study	Predictors	N	Outcome	AUC/C-Stat
Sambarey 2024 [39]	203 multimodal (clinical, imaging, microbiological, genomic)	5060	Treatment outcome	0.84
Zhan 2023 [36]	10 haematological + cavity + smoking	478	Treatment outcome	0.77–0.80
Ma 2023 [38]	health education, age, sex, lung extent	446	Unsuccessful outcome	0.76
Alene 2020 [37]	2nd-line history, FQ resistance, 2-mo smear	570	Poor outcome	0.67–0.69
Present study	3 biomarkers + cavity/radiology (compact)	155	2-month culture positivity	0.70 ± 0.09 (fully nested); 0.74 ± 0.08/0.75 (conditional)

## Data Availability

The de-identified analytic dataset, data dictionary and variable definitions, complete analysis code, random-seed specification, exact resampling specifications, full fitted coefficient table and score-to-probability table are available from the corresponding author upon reasonable request, subject to institutional and GDPR constraints.

## Supplementary Materials

The following supporting information can be downloaded at: https://www.mdpi.com/article/10.3390/life16081384/s1, Table S1: Three-way cross-tabulation of radiological pattern, cavity number and cavity size; Table S2: Complete fitted penalised-logistic model; Table S3: Score-to-probability mapping over the full observed score range; Table S4: Sensitivity harmonization of the two-cavity number/cavity size discordances.
