# Supplementary material for: New Strategies in Tuberculosis Prediction: TB-CAST, an Explainable Baseline Score for Two-Month Sputum Culture Positivity
Source: Life (Basel). 2026 Aug 21;16(8):1384. doi: 10.3390/life16081384 (PMC13514189; doi:10.3390/life16081384)
Supplement: Supplementary file 1 [file life-16-01384-s001.zip › life-4474531-supplementary.pdf]

Supplementary Materials

New Strategies in Tuberculosis Prediction: TB-CAST, an Explainable Baseline Score for Two-Month Sputum Culture Positivity

Table S1. Three-way cross-tabulation of radiological pattern, cavity number and cavity size

Radiological pattern	Cavity number	No cavities	<10 mm	10–25 mm	>25 mm	Total
Infiltrative–nodular	No cavities	12	0	0	0	12
Infiltrative–nodular	One cavity	0	0	0	1	1
Infiltrative–nodular	Two cavities	0	0	0	0	0
Infiltrative–nodular	>2 cavities	0	0	2	2	4
Infiltrative–ulcerative	No cavities	22	1	0	0	23
Infiltrative–ulcerative	One cavity	0	0	0	0	0
Infiltrative–ulcerative	Two cavities	0	0	0	1	1
Infiltrative–ulcerative	>2 cavities	0	0	1	1	2
Cavitary	No cavities	3	0	0	0	3
Cavitary	One cavity	0	0	0	1	1
Cavitary	Two cavities	0	0	1	1	2
Cavitary	>2 cavities	0	0	3	2	5
Mixed	No cavities	23	0	0	1	24
Mixed	One cavity	0	1	5	15	21
Mixed	Two cavities	0	0	1	4	5
Mixed	>2 cavities	0	2	8	41	51

Note. The two source-coded logical cavity number/cavity size discordances are: (i) one infiltrative–ulcerative record coded as "no cavities" by cavity number and "<10 mm" by cavity size; and (ii) one mixed-pattern record coded as "no cavities" by cavity number and ">25 mm" by cavity size. The primary dataset was not overwritten because the source imaging needed for adjudication was not retained in the locked analytic files.

Table S2. Complete fitted penalised-logistic model

Term	Definition	Coefficient β	Integer points	Reference category
Intercept	Intercept	−1.8339	—	—
LEUI	Leukocytes ≥ 9,380/μL	+0.7184	+1	—
RDWsI	RDW-SD ≥ 36 fL	+1.1142	+2	—
MONI	Monocytes ≥ 690/μL	+0.4470	+1	—
NRCAVITATI_2	One cavity	+0.1319	0	No cavities
NRCAVITATI_3	Two cavities	−0.2335	0	No cavities
NRCAVITATI_4	Multiple cavities (>2)	+0.6142	+1	No cavities
DIMENSIUNECAVITATI_1	No cavities	−0.5717	−1	>25 mm
DIMENSIUNECAVITATI_2	Cavity <10 mm	+0.0559	0	>25 mm
DIMENSIUNECAVITATI_3	Cavity 10–25 mm	+0.2115	0	>25 mm
FORMERADIOLOGICE_1	Infiltrative–nodular pattern	+0.3296	+1	Mixed
FORMERADIOLOGICE_2	Infiltrative–ulcerative pattern	−1.0303	−2	Mixed
FORMERADIOLOGICE_3	Cavitary pattern	−0.6783	−1	Mixed

Note. There are 12 fitted predictor terms plus the intercept (13 estimated parameters). L2 penalty, C = 0.5, lbfgs solver, random seed 42. Integer points were obtained by dividing β by the fixed 0.5 log-odds unit and rounding to the nearest integer. Continuous-biomarker indicators used cut-points of 9,380/μL for leukocytes, 36 fL for RDW-SD and 690/μL for monocytes.

Table S3. Score-to-probability mapping over the full observed score range

TB-CAST score	Predicted probability of two-month culture positivity
−3	0.007
−2	0.016
−1	0.036
0	0.078
1	0.163
2	0.308
3	0.505
4	0.700
5	0.843

Note. Probability mapping: logit[P(positive)] = −2.4679 + 0.8291 × score. Values are derivation-cohort model probabilities and require external validation.

Table S4. Sensitivity harmonization of the two-cavity number/cavity size discordances

Metric	Original source coding	Sensitivity harmonisation	Difference
Mean test-fold AUC	0.7394	0.7377	−0.0018
Test-fold AUC SD	0.0762	0.0761	−0.0001
Patient-level OOF score AUC	0.7536	0.7504	−0.0032
Patient-level OOF probability AUC	0.7530	0.7506	−0.0024
Brier score	0.2002	0.2012	+0.0011
≥2: sensitivity / specificity	0.903 / 0.388	0.903 / 0.390	0.000 / +0.002
≥3: sensitivity / specificity	0.803 / 0.596	0.811 / 0.598	+0.008 / +0.002
≥4: sensitivity / specificity	0.569 / 0.737	0.563 / 0.732	−0.006 / −0.004
Rounded integer-point assignments	Unchanged	Unchanged	No change

Note. Sensitivity harmonisation set cavity size to "no cavities" in the two records coded as "no cavities" by cavity number but assigned a non-zero cavity size category. The full conditional fixed-candidate procedure was rerun. This check did not alter the primary locked dataset and does not substitute for adjudication against source images.
